# Enteric viruses in HIV-1 seropositive and HIV-1 seronegative children with diarrheal diseases in Brazil

**DOI:** 10.1371/journal.pone.0183196

**Published:** 2017-08-30

**Authors:** Silvana Augusta Rodrigues Portes, Filipe Anibal Carvalho-Costa, Monica Simões Rocha, Tulio Machado Fumian, Adriana Gonçalves Maranhão, Rosane Maria de Assis, Maria da Penha Trindade Pinheiro Xavier, Myrna Santos Rocha, Marize Pereira Miagostovich, José Paulo Gagliardi Leite, Eduardo de Mello Volotão

**Affiliations:** 1 Laboratory of Comparative and Environmental Virology, Oswaldo Cruz Institute, Oswaldo Cruz Foundation, Rio de Janeiro, Rio de Janeiro, Brazil; 2 Escritório Regional Fiocruz Piauí, Teresina, Piauí, Brazil; 3 Hospital Municipal Jesus, Rio de Janeiro, Brazil; Kliniken der Stadt Köln gGmbH, GERMANY

## Abstract

Diarrheal diseases (DD) have distinct etiological profiles in immune-deficient and immune-competent patients. This study compares detection rates, genotype distribution and viral loads of different enteric viral agents in HIV-1 seropositive (n = 200) and HIV-1 seronegative (n = 125) children hospitalized with DD in Rio de Janeiro, Brazil. Except for group A rotavirus (RVA), which were detected through enzyme immunoassay, the other enteric viruses (norovirus [NoV], astrovirus [HAstV], adenovirus [HAdV] and bocavirus [HBoV]) were detected through PCR or RT-PCR. A quantitative PCR was performed for RVA, NoV, HAstV, HAdV and HBoV. Infections with NoV (19% vs. 9.6%; p<0.001), HBoV (14% vs. 7.2%; p = 0.042) and HAdV (30.5% vs. 14.4%; p<0.001) were significantly more frequent among HIV-1 seropositive children. RVA was significantly less frequent among HIV-1 seropositive patients (6.5% vs. 20%; p<0.001). Similarly, frequency of infection with HAstV was lower among HIV-1 seropositive children (5.5% vs. 12.8%; p = 0.018). Among HIV-1 seropositive children 33 (16.5%) had co-infections, including three enteric viruses, such as NoV, HBoV and HAdV (n = 2) and NoV, HAstV and HAdV (n = 2). The frequency of infection with more than one virus was 17 (13.6%) in the HIV-1 negative group, triple infection (NoV + HAstV + HBoV) being observed in only one patient. The median viral load of HAstV in feces was significantly higher among HIV-1 positive children compared to HIV-1 negative children. Concerning children infected with RVA, NoV, HBoV and HAdV, no statistically significant differences were observed in the medians of viral loads in feces, comparing HIV-1 seropositive and HIV-1 seronegative children. Similar detection rates were observed for RVA, HAstV and HAdV, whilst NoV and HBoV were significantly more prevalent among children with CD4^+^ T lymphocyte count below 200 cells/mm^3^. Enteric viruses should be considered an important cause of DD in HIV-1 seropositive children, along with pathogens more classically associated with intestinal infections in immunocompromised hosts.

## Introduction

Diarrheal diseases (DD) represent one of the leading causes of mortality in children, accounting for almost 10% of deaths in this age group [1]. Viruses are among the most frequently enteric pathogens identified in children with DD worldwide [2]. Classic viral enteropathogens include group A rotaviruses (RVA), noroviruses (NoV), astrovirus (HAstV) and enteric adenovirus (HAdV-F). More recently, emerging agents such as bocavirus (HBoV) and Aichi virus (AiV) have been considered as potential etiological agents of DD [3–5].

RVA (Reoviridae family) are the major etiological agents associated with severe DD in children younger than 5 years of age in developed and developing countries [6]. RVA have been classified into 27 G genotypes and 37 P genotypes based on the nucleotide sequences of the VP7 (G-type) and VP4 (P-type) encoded genes. Combinations of G1, G2, G3, G4, G9, and G12 with P[4], P[6] and P[8] have been the most frequently detected in humans [7].

NoV (Caliciviridae family) are responsible for outbreaks and sporadic cases of DD in all age groups, accounting for 50% of all cases and more than 90% nonbacterial DD outbreaks [8]. NoV were classified into seven genogroups (GI to GVII) [9,10]. NoV GI, GII and GIV infect humans, with at least 36 genotypes described so far [8, 11, 12]. The NoV GII is the most frequently detected worldwide, with GII.4 being the most prevalent in DD [10].

HAstV are considered important etiological agents associated with DD in children under 5 years [13]. They belong to family Astroviridae and genus *Mamastrovirus* (MAstV 1- classical human astrovirus 1–8) and are often detected in children with DD, with HAstV-1 being most commonly detected [14].

HAdV are frequently detected in outbreaks and sporadic DD in children under 5 years [15, 16]. HAdV belong to Adenoridae family, *Mastadenovirus* genus and are classified into seven species of HAdV (HAdV-A to -G) with a total of 78 types of HAdV reported. HAdV are associated with different syndromes such as respiratory infections, conjunctivitis and DD. Enteric HAdV-F40 and F41 (species F) are the third most common cause of non-bacterial diarrhea among children. Other species such as A, B, C, G and D have also been detected in DD [17].

Among emerging viral enteric pathogens, HBoV (*Parvoviridae* family) is a small non-enveloped single-stranded DNA virus identified in 2005 and proposed initially as a putative agent of acute respiratory tract infections [18]. HBoV has also been identified in human stool samples [19] and, in patients with DD, usually present in co-infections with other viral pathogens such as RVA, NoV, and HAstV [20, 21]. Four genotypes have been described: HBoV1 in respiratory samples and HBoV2, HBoV3 and HBoV4 in fecal samples [4, 19, 22].

DD is a very frequent clinical complication and a common cause of hospitalization and death among human immunodeficiency virus 1 (HIV-1) seropositive children [23, 24, 25]. Concerning enteric viruses, different agents have been identified in association with DD in HIV-1 seropositive patients [26, 27, 28, 29]. The positivity for at least one viral agent in HIV-1 seropositive subjects with DD ranges between 6.4% and 52% in distinct surveys carried out in the United States and South America [23, 30, 31, 32].

DD has distinct etiological profiles in immune-deficient and immune-competent patients. In this context it was observed that the detection rate of RVA in children with DD is higher in HIV negative than in HIV positive children in Tanzania [33]. This contrasts with a higher positivity for caliciviruses among HIV-1 positive children when compared to HIV-1 negative children, as described in Venezuela [34].

In HIV-1 positive adults with DD in Brazil, agents such as herpes simplex virus 1 and 2 (HSV-1/2), cytomegalovirus (CVM), HAdV and emerging viruses such as HBoV have been identified; these viruses frequently co-infect patients harboring parasites, including *Isospora belli*, *Giardia duodenalis*, *Strongyloides stercoralis* and *Entamoeba histolytica/Entamoeba dispar* [35].

The objective of this study was to compare detection rates, genotype distribution and viral load of different enteric viral agents in HIV-1 seropositive and HIV-1 seronegative children hospitalized with DD in Rio de Janeiro, Brazil.

## Materials and methods

### Ethical statement

This study was approved by the Ethics Committee of the Oswaldo Cruz Foundation (CEP No. 311/06) and is part of an official surveillance of the Brazilian Ministry of Health (MS, Portuguese acronym for Ministério da Saúde) of enteric pathogens to investigate the viral etiology of DD. This surveillance is performed through a hierarchical network in which the samples are provided by medical request in hospitals and health centers, monitored by Unified Health System (SUS, Portuguese acronym for Sistema Único de Saúde). Fecal samples were sent to the Laboratory of Comparative and Environmental Virology (LVCA) of the Oswaldo Cruz Institute (Fiocruz/MS). Records containing epidemiological and clinical data followed each fecal sample. Data are maintained anonymously and securely.

### Setting

The study was performed in Rio de Janeiro, Brazil (population = 6,300,000 inhabitants). The incidence rate of HIV-1 infection is 41.1 new cases/100,000 inhabitants/year; one of the highest in the country. In Brazil, AIDS incidence in children has reduced substantially following implementation of HIV-1 testing during pregnancy, rapid anti-HIV-1 testing in maternity hospitals and the universal access of exposed newborns to antiretroviral prophylaxis. Children were recruited in two public pediatric hospitals: i) Hospital Municipal Jesus (HMJ), a tertiary pediatric center providing specialized medical care and ii) Hospital Municipal Salles Netto (HMSN), a pediatric general hospital. These hospitals are situated downtown in Rio de Janeiro, 6km distant from each other, providing medical care for children from low and middle socioeconomic status. Identification of enteric viruses was carried out at the LVCA (Fiocruz), 10km from both hospitals.

### Study design, case definition and collection of fecal samples

We compared 200 fecal samples representing different hospitalizations of 123 HIV-1 seropositive children in HMJ (so that some children [n = 49] were hospitalized more than once) with 125 fecal samples representing hospitalizations of 125 seronegative children (in this group each children was hospitalized once) between the years 1997 and 2010. HIV-1 seropositive and HIV-1 seronegative children were paired by year. The minimum period between hospitalizations was 2 months. Among the 123 HIV seropositive children, 98 (80%) were under highly active antiretroviral therapy (HAART). Among HIV-1 seropositive children, anti-HIV serological status was confirmed through ELISA and/or Western-blot assays. HIV-1 seropositive and HIV-1 seronegative children were matched by age. Age group distribution among HIV-1 seropositive and HIV-1 seronegative children was as follows: i) less than 25 months, 34% (n = 69) and 49.5% (n = 62); ii) 25 to 60 months, 28.6% (n = 57) and 28% (n = 35); more than 60 months, 37.4% (n = 74) and 22.4% (n = 28), respectively. HIV-1 seropositive children were immunocompromised to various degrees; CD4^+^ T lymphocyte count was assessed in 121 HIV-1 seropositive patients: 38 had ≤ 100 cells/mm^3^, 11 had 100–200 cells/mm^3^, 13 had 201–350 cell/mm^3^, 6 had 351–500 cells/mm^3^ and 53 had > 500 cells/mm^3^.

Children recruited were participants in a project of DD etiological monitoring conducted in both hospitals during the study period. DD was defined by liquid or semi-liquid stools (with or without fever, vomiting, and abdominal pain) associated with dehydration and the necessity of intravenous fluid replacement. Liquid or semiliquid fecal samples were collected in plastic bottles by health personnel during hospitalization, and stored briefly in the hospital at 4°C until transport to the LVCA, where they were transferred to freezers at -20°C for viral identification.

### Statistical analysis

The positivity rates for distinct enteric viruses in HIV-1 seropositive and HIV-1 seronegative children were compared through the Fisher’s exact test (two-tailed). Viral loads among HIV-1 seropositive and HIV-1 seronegative patients, as well as among HIV-1 seropositive children with CD4^+^ T lymphocyte count below and above 200 cells/mm^3^ were compared with the Kruskal-Wallis test. Statistical significance was established at p<0.05.

### Nucleic acid purification, detection and characterization of enteric viruses

Viral nucleic acids were purified from stool samples stored at –20°C. 140μL fecal suspensions (10%v/v) were prepared in Tris-calcium buffer (Tris/HCl/Ca2+, pH 7.2). The extraction of viral DNA and RNA was performed using the methodology of the QIAamp Viral RNA Mini Kit (QIAGEN^®^, Valencia, CA, USA). RNA was transcribed to complementary DNA (cDNA) using the High Capacity cDNA Reverse Transcription kit (Applied Biosystems, Foster City, CA, USA) according to the manufacturer’s instructions. Aliquots were immediately stored at -80°C. In each extraction procedure RNAse/DNAse-free water was used as negative control.

Except for RVA, which were detected through enzyme immunoassay kits (EIARA^®^, Biomanguinhos, Rio de Janeiro, Rio de Janeiro, Brazil; Premier Rotaclone^®^, Meridian Bioscience Inc, Cincinatti, Ohio, USA or Ridascreen Rotavirus^®^, R-Biopharm, Darmstadt, Hesse, Germany) and polyacrylamide gel electrophoresis (PAGE) [36], and genotyped by semi-nested multiplex reverse transcription polymerase chain reaction (RT-PCR), the other enteric viruses (NoV, HAstV, HAdV and HBoV) were detected though PCR or RT-PCR with sets of primers that amplify specific regions used for viral detection and characterization (Table 1). PCR amplicons were purified using QIAquick Gel Extraction Kit and PCR Purification Kit (Qiagen, Inc., Valencia, CA, USA) following the manufacturer’s recommendations. The purified DNA amplicons were sequenced using the BigDye^®^ Terminator v3.1 Cycle Sequencing Kit and ABI Prism 3100 Genetic Analyser sequencer (both from Applied Biosystems, Foster City, CA, USA). Sequences were edited and aligned using BioEdit Sequence Alignment Editor Program (Hall, 1999) and subsequently compared to those available in GenBank database using the Basic Local Alignment Search Tool (BLAST). For NoV, genotyping was assigned using an online genotyping tool (http://www.rivm.nl/mpf/norovirus/typingtool) and the strains were named, with the genotype of the polymerase indicated with an uppercase letter p [11,12].

**Table 1:** Oligonucleotide primers and probes used for viral detection, quantification and molecular characterization.

Virus	Primer	Genomic region	References
RVA^a^	9con1,9con2, 9T1-1 (G1), 9T1-2 (G2), 9T1-3P (G3), 9T1-4 (G4), 9T-9B (G9), FT5 (G5)	VP7	[36,37,38,39,40]
	4con2, 4con3, 1T1, 1T1-Wa, 1T1-VN P[8], 2T1 P[4], 3T1 P[6], 4T1 P[9], 5T1 P[5]	VP4	
RVA^b^	NSP3F, NSP3R, NSP3P	NSP3	[41]
NoV^a^	GI: Mon 432, Mon 434	Region B (polymerase)	[42]
	GII: Mon 431, Mon 433		
	GI: Cap A, CapB1, CapB3	Region D (Capsid)	[43]
	GII:Cap C, CapD1, CapD3		
	GII: Mon431, G2SKR	ORF1-2 junction	[42,44]
	GII: COG2F, G2SKR	5’ORF2 junction	[44,45]
NoV^b^	GI: COG 1, COG1R, RING1cP	ORF1-2 junction	[45]
	GII: COG 2F, COG2R, RING2P		
HAstV^a^	Mon 269, Mon 270	ORF-2	[46]
HAstV^b^	AstVF, AstVR, AstVP	ORF1b-ORF2 junction	[47]
HAdV^a^	Hex1deg, Hex2deg	Hexon	[48]
HAdV^b^	AdF, AdR, Adp1	Hexon	[49]
HBoV^a^	AK-VP-F1, AK-VP-R1	VP1/2	[22]
	Ak-VP-F2, AK-VP-R2		
HBoV^b^	HoV1F, HBoV1R, HBoV234F, HBoV 24R, HBoV3R, H1234probe	UTR-NS1 junction	[50]

A rotavirus (RVA), norovirus (NoV), human adenovirus (HAdV), human astrovirus (HAstV) and human bocavirus (HBoV).

^*a*^Primes used for molecular detection and characterization.

^*b*^ Primes and probes used for detection and quantification (qPCR).

The nucleotide sequences obtained in this study were submitted to the NCBI (GenBank, http://www.ncbi.nlm.nih.gov/) and received accession numbers: KY611586-KY611611; MF150192-MF150203; KY882298-KY882312; KY910901-KY910941; KY753477-753502; MF034109-MF034119; MF034120-MF034125; MF156863-MF156875; MF156863-MF156866; MF156867-MF156875.

### Quantification of viral loads

A quantitative PCR (qPCR) was performed for detection and quantification of RVA, NoV, HAstV, HAdV and HBoV according to previously described protocols (Table 1). All qPCR reactions were carried out in an ABI PRISM 7500^®^ Real-Time System v2.0 (Applied Biosystems, Foster City, CA). All assays were performed with TaqMan universal master mix^®^ (Applied Biosystems, Foster City, CA). Undiluted and 10-fold dilutions of the nucleic acid and cDNA were analyzed in duplicated and concentrations were estimated as the mean of data obtained, correcting for the dilution analyzed. Amplifications were performed in a thermocycler programmed as follows: incubation at 50°C for 2 min to activate UNG, initial denaturation at 95°C for 10 min, followed by 45 cycles at 95°C for 15s and 60°C for 1min. The negative control used was a PCR TaqMan master mix without DNA.

## Results

### Detection rates of enteric viruses and genotype distribution in HIV-1 seropositive and HIV-1 seronegative patients

Among HIV-1 seropositive children, 148/200 (74%) were infected with at least one virus, versus 80/125 (64%) among HIV-1 seronegative children. As presented in Table 2, infections with NoV (19% vs. 9.6%; p<0.001), HBoV (14% vs. 7.2%; p = 0.042) and HAdV (30.5% vs. 14.4%; p<0.001) were significantly more frequent among HIV-1 seropositive children. On the other hand, RVA was significantly less frequent among HIV-1 seropositive patients with DD (6.5% vs. 20%; p<0.001). Similarly, frequency of infection with HAstV was lower among HIV-seropositive children (5.5% vs. 12.8%; p = 0.018).

**Table 2:** Enteric virus detection in HIV-1 seropositive children and HIV-1 seronegative children hospitalized with diarrheal diseases by age group in Rio de Janeiro, Brazil.

	Fecal samples from 123 HIV-1 seropositive children (n = 200 samples)	Fecal samples from 125 HIV-1 seronegative children (n = 125 samples)	p-value^a^
*Virus/Age group*			
**Group A rotavirus**			
0–24 months	10/69 (14.5%)	21/62 (33.9%)	0.008
25–60 months	0/57 (0%)	4/35 (11.4%)	0.018
>60 months	3/74 (4.1%)	0/28 (0%)	0.377
Total	13/200 (6.5%)	25/125 (20%)	<0.001
**Norovirus**			
0–24 months	9/69 (13%)	5/62 (8.1%)	0.263
25–60 months	7/57 (12.3%)	4/35 (11.4%)	0.589
>60 months	19/74 (25.7%)	3/28 (10.7%)	0.081
Total	38/200 (19%)	12/125 (9.6%)	0.033
**Human astrovirus**			
0–24 months	4/69 (5.8%)	11/62 (17.7%)	0.030
25–60 months	3/57 (5.3%)	3/35 (8.6%)	0.413
>60 months	4/74 (5.4%)	2/28 (7.1%)	0.527
Total	11/200 (5.5%)	16/125 (12.8%)	0.018
**Human bocavirus**			
0-24months	11/69 (15.9%)	5/62 (8.1%)	0.133
25–60 months	7/57 (12.3%)	1/35 (2.9%)	0.117
>60 months	10/74 (13.5%)	3/28 (10.7%)	0.497
Total	28/200 (14%)	9/125 (7.2%)	0.042
**Human adenovirus**			
0–24 months	16/69 (23.2%)	13/62 (21%)	0.463
25–60 months	27/57 (47.4%)	4/35 (11.4%)	<0.001
>60 months	18/74 (24.3%)	1/28 (3.6%)	0.011
Total	61/200 (30.5%)	18/125 (14.4%)	<0.001

^a^Fisher`s exact test.

Table 3 presents genotype distribution of different enteric viruses in HIV-1 seropositive and HIV-1 seronegative patients. It was observed that, among children infected with RVA, G1, G2, G3 and G9 genotypes were detected in HIV-1 seropositive, while G1, G2, G3, G4, G5 and G9 genotypes were in HIV-1 seronegative children. The P[4] and P[8] genotypes were detected in HIV-1 seropositive and seronegative subjects. The combinations G1P[8] and G9P[8] predominated in both groups. Among children in whom NoV was detected, a great diversity of genotypes was observed. Genogroup II was the most frequently identified both in HIV-1 seropositive and HIV-1 seronegative patients, with a predominance of GII.4 and GII.12 in HIV-1 seropositive and GII.4 in HIV-1 seronegative children. GII.4 variants detected were Den Haag_2006b and US95_96 among HIV-1 seropositive children whereas among HIV-1 seronegative children were Kaiso_2003, Asia_2003 and Den Haag_2006b. NoV recombinant genotypes GII.Pa-GII.3, GII.P12-GII10 and GII.P7-GII.6 were detected among HIV-1 seropositive children, with GII.P7-GII.6 being the most detected. HAstV genotype 1 was the most frequent both in HIV-1 seropositive and HIV-1 seronegative subjects. In HIV-1 seronegative patients, HAstV 2 was the second most detected virus followed by HAstV3. HBoV genotype 1 was the most prevalent in both groups and genotype 2 the second most detected in HIV-1 seropositive children. Among HAdV positive patients, non-enteric species (A, B, C or D) were detected frequently in diarrheic feces from HIV-1 seropositive (25/61 [41%]) and HIV-1-seronegative (9/18 [50%]) children. HAdV D was the second most detected among HIV-1 seropositive children losing only to HAdV- F40. Concerning enteric HAdV types, F40 was detected more frequently than F41 in both groups. All not typed samples presented low viral load by qPCR and failed to attempt genotyping by PCR.

**Table 3:** Genotype distribution of different enteric viruses in fecal samples obtained from 123 HIV-1 seropositive children (n = 200 fecal samples) and 125 HIV-1 seronegative children ((n = 125 fecal samples) hospitalized with diarrheal diseases in Rio de Janeiro, Brazil.

	Fecal samples obtained from HIV-1 seropositive children	Fecal samples obtained from HIV-1 seronegative children
**Group A rotavirus genotypes**	**n = 13**	**n = 25**
G1P[8]	4 (30.8%)	8 (32%)
G2P[4]	3 (23.1%)	1 (4%)
G2P[8]	0 (0%)	1 (4%)
G3P[NT]	0 (0%)	1 (4%)
G3P[8]	1 (7.7%)	0 (0)
G4P[8]	0 (0%)	1 (4%)
G5P[8]	0 (0%)	2 (8%)
G9P[8]	5 (38.5%)	11 (44%)
**Norovirus genotypes**	**n = 38**	**n = 12**
GI (not typed)	1 (2.6%)	1 (8.3%)
GI.2	1 (2.6%)	0 (0%)
GI.3	1 (2.6%)	0 (0%)
GII (not typed)	6 (15.8%)	3 (25%)
GII.2	3 (7.9%)	0 (0%)
GII.3	1 (2.6%)	0 (0%)
GII.4 US95_96	1 (2.6%)	0 (0%)
GII.4 Kaiso_2003	0 (0%)	3 (25%)
GII.4 Asia_2003	0 (0%)	2 (16.7%)
GII.4 Den_Haag_2006b	1 (2.6%)	2 (16.7%)
GII.6	3 (7.9%)	0 (0%)
GII.7	0 (0%)	1 (8,3%)
GII.10	1 (2.6%)	0 (0%)
GII.12	6 (15.8%)	0 (0%)
GII.14	1 (2.6%)	0 (0%)
GII.Pa-GII.3	1 (2.6%)	0 (0%)
GII.P4-GII.4	1(2.6%)	0 (0%)
GII.P4 US95_96-GII.4	1 (2.6%)	0 (0%)
GII.P4-GII.4 US95_96	2 (5.3%)	0 (0%)
GIIP7-GII.6	4 (10.5%)	0 (0%)
GII.P7-GII.7	2 (5.3%)	0 (0%)
GII.P12-GII.10	1 (2.6%)	0 (0%)
**Human astrovirus genotypes**	**n = 11**	**n = 16**
1	10 (90.9%)	8 (50%)
2	0 (0%)	3 (18.7%)
3	0 (0%)	2 (12.5%)
Not typed	1 (9.1%)	3 (18.7%)
**Human bocavirus genotypes**	**n = 28**	**n = 9**
1	14 (50%)	6 (66.7%)
2	8 (28.6%)	1 (11.1%)
3	3 (10.7%)	1 (11.1%)
4	1 (3.6%)	0 (0%)
Not typed	2 (7.1%)	1 (11.1%)
**Human adenovírus genotypes**	**n = 61**	**n = 18**
A-12	3 (4,9%)	1 (5,6%)
A-31	0 (0%)	1 (5,6%)
B-3	2 (3,3%)	0 (0%)
B-7	1 (1,6%)	2 (11,1%)
C-1	2 (3,3%)	0 (0%)
C-2	6 (9,8%)	3 (16,7%)
C-6	1 (1,6%)	0 (0%)
D	10 (16,4%)	2 (11,1%)
F-40	30 (49,2%)	7 (38,9%)
F-41	6 (9,8%)	2 (11,1%)

### Multiple viral agents infecting HIV-1 seropositive and HIV-1 seronegative children

As presented in Table 4, 33/200 (16.5%) HIV-1 seropositive patients were excreting more than one enteric virus in fecal samples. In this group, co-infections with three enteric viruses, such as NoV, HBoV and HAdV (n = 2) and NoV, HAstV and HAdV (n = 2) were identified. The rate of infection with more than one virus was 17/125 (13.6%) in the HIV-1 seronegative group, triple infection (NoV + HAstV + HBoV) being observed in only one patient.

**Table 4:** Detection of multiple enteric viruses in fecal samples from 123 HIV-1 seropositive children (n = 200 fecal samples) and 125 HIV-1 seronegative children ((n = 125 fecal samples) hospitalized with diarrheal diseases in Rio de Janeiro, Brazil.

No. of viruses	Virus combination in fecal samples	Fecal samples from 123 HIV-1 seropositive children (n = 200 samples)	Fecal samples from 125 HIV-1 seronegative children (n = 125 samples)
**Two**	HAstV + HBoV	1 (0.5%)	1 (0.8%)
	HAstV + HAdV	4 (2%)	3 (2.4%)
	HBoV + HAdV	6 (3%)	0 (0%)
	NoV + HAstV	0 (0%)	1 (0.8%)
	NoV + HBoV	5 (2.5%)	1 (0.8%)
	NoV + HAdV	6 (3%)	2 (1.6%)
	RVA + HAstV	0 (0%)	4 (3.2%)
	RVA + HBoV	3 (1.5%)	1 (0.8%)
	RVA + HAdV	1 (0.5%)	2 (1.6%)
	RVA + NoV	2 (1%)	1 (0.8%)
	Total no. of double infections (%)	28 (14%)	16 (12.8%)
**Three**			
	HAstV + HBoV + HAdV	1 (0.5%)	0 (0%)
	NoV + HAstV + HBoV	0 (0%)	1 (0.8%)
	NoV + HAstV + HAdV	2 (1%)	0 (0%)
	NoV + HBoV + HAdV	2 (1%)	0 (0%)
	Total no. of triple infections (%)	5 (2,5%)	1 (0.8%)

Rotavirus A (RVA), norovirus (NoV), human adenovirus (HAdV), human astrovirus (HAstV) and human bocavirus (HBoV);

### Viral load of enteric viruses in HIV-1 seropositive and HIV-1 seronegative children

**Fig 1:**
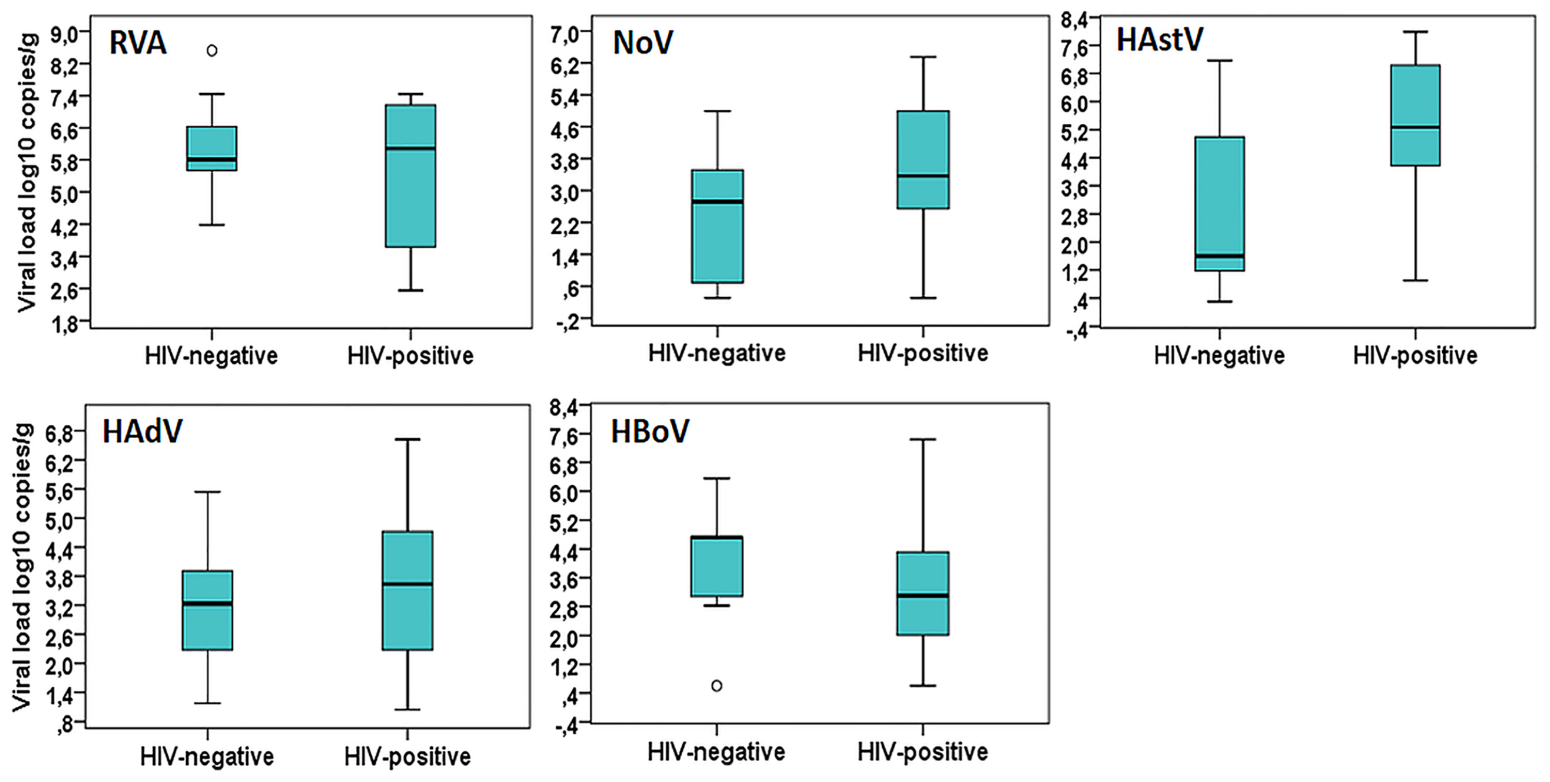
Fecal viral loads of enteric viruses in HIV-1 seropositive and seronegative children hospitalizaded with diarrheal diseases in Rio de Janeiro, Brazil.

As demonstrated in Fig 1, the median viral load of HAstV in feces was significantly higher among HIV-1 seropositive children compared to HIV-1 seronegative children (p = 0.012). Concerning children infected with RVA, NoV, HBoV and HAdV, no statistically significant differences were observed in the medians of viral loads in feces when comparing HIV-1 seropositive and HIV-1 seronegative children. Comparing viral loads of distinct enteric viruses in HIV-1 seronegative patients, it was observed that RVA presented the higher median (5.81 [IQR = 5.53–6.62] log10 copies/g). In this group, median viral load in feces for NoV, HAstV, HBoV and HAdV reached 2.72 (IQR = 0.69–3.51), 1.29 (IQR = 1.17–4.99), 4.72 (IQR = 3.07–4.72), and 3.23 (IQR = 2.27–3.90) log10 copies/g, respectively. RVA also presented the higher median viral load among HIV-1 seropositive children: 6.08 (IQR = 3.36–7.17) log10 copies/g. Among HIV-1 seropositive patients, viral loads in feces were as follows: NoV, 3.36 (IQR = 2.54–4.99), HAstV, 5.26 (4.18–7.43), HBoV, 3.09 (2–4.31), HAdV, 3.63 (2.27–4.72) log10 copies/g. In HIV-1-seropositive patients, median viral loads of enteric (types F-40 and F-41) and non-enteric (types A-12, A-31, B-3, B-7, C-1, C-2, C-6, and D) HAdV were similar (3.63 [IQR = 2.73–4.53) vs. 3.24 [IQR = 2–5.28], p = 0.210) (not shown).

### Frequency of infection with enteric viruses and viral loads by level of CD4^+^ T lymphocyte count

From 200 HIV-1 seropositive, DD-related hospitalizations, we could assess CD4^+^ T lymphocyte count in 121 children. From these, 49 had <200 cells/mm^3^ and 72 presented a count ≥200 cells/mm^3^. It was examined whether among HIV-1 seropositive children, those with a CD4^+^ T lymphocyte count below 200 cells/mm^3^ had a different enteric virus profile than children with a count above 200 cells/mm^3^. Similar detection rates were observed for RVA, HAstV and HAdV, whilst NoV and HBoV were significantly more frequent among children exhibiting a CD4^+^ T lymphocyte count below 200 cells/mm^3^.

Differences in viral loads in both groups were not statically significant for distinct viruses (Table 5).

**Table 5:** Rate of detection and fecal viral load of different enteric virus in fecal samples obtained from HIV-1 seropositive children by level of CD4^+^ T lymphocyte count in Rio de Janeiro, Brazil.

	CD4 T lymphocytes < 200 cells/*mm*^3^ (n = 49 fecal samples)	CD4 T lymphocytes ≥ 200 cells/*mm*^3^ (n = 72 fecal samples)	*P*-value
***Group A rotavirus***			
Rate of detection; No. Positive / No. tested (% positive)	4 (8.2%)	3 (4.2%)	0.439^a^
Median viral load in log10 copies/g (IQR)	6.62 (5.81–7.30)	6.08 (3.63–7.16)	0.471^b^
***Norovirus***			
Rate of detection; No. Positive / No. tested (% positive)	18 (36.7%)	8 (11.1%)	0.001^a^
Median viral load in log10 copies/g (IQR)	3.90 (2.54–4.99)	2.82 (1.72–5.26)	0.367^b^
***Human astrovirus***			
Rate of detection; No. Positive / No. tested (% positive)	0 (0%)	4 (5.6%)	0.146^a^
Median viral load in log10 copies/g (IQR)	-	4.72 (3.23–6.62)	-
***Human bocavirus***			
Rate of detection; No. Positive / No. tested (% positive)	12 (24.5%)	7 (9.7%)	0.041^a^
Median viral load in log10 copies/g (IQR)	3.09 (2.27–4.18)	2.82 (1.46–5.53)	0.831^b^
***Human adenovirus***			
Rate of detection; No. Positive / No. tested (% positive)	13 (26.5%)	26 (36.1%)	0.321^a^
Median viral load in log10 copies/g (IQR)	3.36 (1.73–4.36)	4.01 (3.27–4.89)	0.189^b^

^a^Fisher`s exact test

^b^Kruskal-Wallis test

## Discussion

The present study explores the diversity of viral agents in HIV-1 seropositive patients hospitalized with DD, comparing their etiological profile with a group of HIV-1 seronegative children. In this sense, the initial findings were significantly higher NoV, HBoV and HAdV detection rates in the HIV-1 seropositive group. RVA and HAstV were more frequent among HIV-1 seronegative children. In the present study, the opportunistic character of NoV may be evidenced by the fact that, among HIV-1 seropositive children, its positivity rate was more than 3 times higher among patients with CD4 T lymphocyte counts <200 cells/mm3. Despite viral loads being higher among HIV-1 seropositive children, and particularly among those more severely immunocompromised, differences were not statistically significant. In Brazil, NoV has been associated with DD in HIV-1 positive patients [29].

Our results confirm the detection of different genotypes in this group as well as demonstrating the high genetic diversity among HIV-1 seropositive children. Recombinant NoV circulation has been observed when the combined characterization of both regions of the polymerase and capsid are used. Using both regions we characterized recombinant strains (GII.Pa-GII.3, GII.P12-GII10 and GII.P7-GII.6) for the first time in HIV-1 seropositive children in Brazil. The high genetic diversity of NoV has been described among HIV-1 seronegative individuals [51]. Our data reaffirm that NoV is an important agent of DD in HIV-1 seropositive children in Brazil. NoV has been associated with DD in HIV positive patients, and in some studies it has been detected more frequently in this group, as demonstrated in Tanzania [52]. The importance of NoV as a cause of hospitalization and DD-associated death in HIV-1 seropositive children was also demonstrated in South Africa [53]. In Venezuela it was observed that NoV excretion is significantly more frequent in HIV-1 seropositive children, regardless of whether or not they are suffering from DD [34]. Frequent NoV infection in HIV-1 seropositive children with and without DD has also been reported in Kenya in a study that points to the high genetic diversity of NoV in HIV-1 seropositive children [54].

The present study suggests that HBoV is excreted more frequently in HIV-1 seropositive children than in HIV-1 seronegative children. Cases of HBoV co-infections with other enteric viral agents were common and the most frequent HBoV genotypes identified were 1, 2 and 3. Interestingly, among HIV-1 seropositive children HBoV, like NoV, was significantly more frequent in the group with CD4 T lymphocyte counts <200 cells/mm3.HBoV is an emerging pathogen, considered as a potential DD agent. Its clinical and epidemiological importance as a DD agent has not yet been fully clarified. HBoV has been identified in respiratory secretion of HIV-1 seropositive and HIV-1 seronegative patients with acute respiratory infections [55, 56] and in the feces of HIV-1 seropositive patients in Brazil [35, 57]. Additional studies should be performed to more accurately characterize the role of HBoV as an opportunistic agent in immunocompromised children.

This study demonstrated that HAdV is detected more frequently in HIV-1 seropositive than in HIV-1 seronegative patients and therefore presents an opportunistic behavior. Studies from Brazil report HAdV excretion by HIV-1 seropositive children with and without DD [58]. HAdV is a common cause of pediatric DD, also causing respiratory infections. In immunocompromised patients, other infections, especially CMV, may coexist with HAdV. HAdV associated colitis is a clinical entity characterized by persistent diarrhea described in HIV-1 seropositive patients in the pre-HAART era [26, 27, 59]. For the first time in Brazil, we are reporting enteric HAdV in HIV-1 seropositive patients. In this study, the enteric HAdV type F-40 was detected in almost one-half of HAdV-1 positive fecal samples. However, a great diversity of types was observed, some not commonly associated with DD. Similar viral loads of enteric and non-enteric HAdV were observed, suggesting comparable replication intensity in the digestive tract. Among HIV-1 seropositive children, HAdV was detected with similar frequency and viral load among patients with CD4^+^ T lymphocyte counts bellow and above <200 cells/mm3.

Results from this study suggest that RVA is a pathogen related neither to HIV-infection, nor to immunodeficiency. RVA is actually a significantly more common pathogen in children with DD hospitalized in a general pediatric hospital with no underlying diseases or complex co-morbidities such as AIDS, as demonstrated in Tanzania [33]. Nevertheless, RVA is also detected in HIV-positive children, particularly among those younger than 24 months. RVA genotype distribution was similar, with predominance of G1P[8] and G9P[8] both in HIV-1 seropositive and HIV-1 seronegative children. RVA is a common cause of DD in pediatric patients, worldwide. In the last decade, its detection rate in children with DD has been reduced due to vaccination with monovalent and pentavalent vaccines in countries such as Brazil, where universal vaccination with the monovalent vaccine has been implemented in 2006 [6]. Therefore, in this study, children recruited until 2006 were not vaccinated with the monovalent rotavirus vaccine. The monovalent rotavirus vaccine is not indicated for immunocompromised HIV-1 seropositive children (CD4^+^ T lymphocyte counts <200 cells/mm^3^). From 2006 to 2010, vaccine coverage ranged from 70% a 90% in Rio de Janeiro, for the cohort of children born after March 2006.

HAstV was also more frequent among HIV-1 seronegative children. Despite this, viral load was significantly higher among HIV-1 seropositive children when infected by HAstV, regardless of CD4^+^ T lymphocyte count. HAstV has been detected in HIV-1 seropositive children [31], but its importance as a DD agent in this group has not been established.

Data from this study demonstrate that viral agents are often found in HIV-1 seropositive children hospitalized with DD. As a limitation of this study we highlight the fact of having analyzed children with severe forms of DD, which motivated the hospitalizations, providing an etiological profile of the viral agents in this segment of patients. However, the circulation of enteric viruses in asymptomatic children has been reported, which is important to the DD dynamic of transmission. Thus, enteric viruses should be considered an important cause of DD in this group, along with pathogens more classically associated with intestinal infections in immunocompromised hosts. Management of hospitalized HIV-1 seropositive children with DD should consider enteric viral agents.
